# Tamarixetin Protects Chondrocytes against IL-1β-Induced Osteoarthritis Phenotype by Inhibiting NF-κB and Activating Nrf2 Signaling

**DOI:** 10.3390/antiox13101166

**Published:** 2024-09-25

**Authors:** Seung-Ho Lee, Min Kyoung Shin, Jung-Suk Sung

**Affiliations:** Department of Life Science, Dongguk University-Seoul, Goyang 10326, Republic of Korea; q969@dongguk.edu (S.-H.L.); shinmk94@dgu.ac.kr (M.K.S.)

**Keywords:** osteoarthritis, tamarixetin, antioxidant, Nrf2, NF-κB, pyroptosis

## Abstract

Osteoarthritis (OA) is a degenerative joint disease characterized by cartilage breakdown and chronic inflammation in joints. As the most prevalent form of arthritis, OA affects around 600 million people globally. Despite the increasing number of individuals with OA risk factors, such as aging and obesity, there is currently no effective cure for the disease. In this context, this study investigated the therapeutic effects of tamarixetin, a flavonoid with antioxidative and anti-inflammatory properties, against OA pathology and elucidated the underlying molecular mechanism. In interleukin-1β (IL-1β)-treated chondrocytes, tamarixetin inhibited the OA phenotypes, restoring cell viability and chondrogenic properties while reducing hypertrophic differentiation and dedifferentiation. Tamarixetin alleviated oxidative stress via the nuclear factor erythroid 2-related factor 2 (Nrf2) pathway activation and inhibited mitogen-activated protein kinase and nuclear factor-κB (NF-κB). Furthermore, tamarixetin attenuated pyroptosis, a programmed cell death caused by excessive inflammation, by suppressing inflammasome activation. We confirmed that the chondroprotective effects of tamarixetin are mediated by the concurrent upregulation of Nrf2 signaling and downregulation of NF-κB signaling, which are key players in balancing antioxidative and inflammatory responses. Overall, our study demonstrated that tamarixetin possesses chondroprotective properties by alleviating IL-1β-induced cellular stress in chondrocytes, suggesting its therapeutic potential to relieve OA phenotype.

## 1. Introduction

Osteoarthritis (OA) is the most prevalent form of arthritis, affecting around 600 million people worldwide [1,2]. Increasing life expectancy and obesity rates are contributing to a growing number of patients and earlier onset of the disease [3,4]. Currently, non-surgical therapies, including pharmacological and rehabilitation treatments, are utilized for mild to moderate OA symptoms, while surgical intervention is considered the last option for severe patients [5]. In terms of pharmacological treatment, only drugs that alleviate inflammation and pain are approved by the United States Food and Drug Administration (FDA) for the treatment of OA [6]. Therefore, there is an urgent need to discover therapeutic remedies that can restore cartilage homeostasis and prevent long-term disability, such as recovering chondrocyte function and ameliorating cellular stress driving OA.

OA is characterized by structural and functional deterioration, along with chronic inflammation in the joint, leading to impaired mobility [7] and inducing severe pain [8]. Cartilage destruction results from injuries or aging of joints, which increases mechanical stress [9] and secretion of pro-inflammatory mediators in the cartilage and synovium [10]. Following the onset of inflammation and oxidative stress, the phenotype of chondrocytes shifts towards hypertrophic differentiation and dedifferentiation [11,12]. Chondrocytes undergoing hypertrophy highly express proteolytic enzymes, including matrix metalloproteinase 1 (MMP1), MMP9, and MMP13. Extracellular matrix (ECM) secretion also becomes altered, with decreased expressions of type II collagen (COL2) and aggrecan (ACAN) decrease and increased expression of type X collagen (COLX) [13]. In addition, the dedifferentiation of chondrocytes also increases the secretion of type I collagen (COL1), which can cause cartilage fibrosis. These dysfunctions of chondrocytes disrupt cartilage homeostasis, resulting in a stiffer composition of the cartilage matrix and increased mechanical stress [14].

The major cause of inflammation and oxidative stress in the cartilage under OA is the presence of damage-associated molecular patterns (DAMPs), including pro-inflammatory cytokines and components of degraded ECM [15]. Among such mediators, interleukin-1 beta (IL-1β) plays a key role in OA pathology [16,17,18]. The binding of IL-1β to the interleukin-1 receptor (IL-1R) leads to the intracellular signaling transduction that activates mitogen-activated protein kinase (MAPK) and nuclear factor-κB (NF-κB) downstream. This aggravates the pathology of OA by inducing inflammatory responses, downregulating anabolism, and upregulating catabolism in chondrocytes [19]. Nuclear factor erythroid 2-related factor 2 (Nrf2) is a master regulator of an antioxidative pathway that concurrently regulates cellular responses to inflammation and oxidative stress in collaboration with NF-κB [20,21]. The complex interplay between these transcriptional factors contributes to the onset and development of OA pathology [22]. The excess generation of reactive oxygen species (ROS) by the IL-1β/IL-1R signaling further exacerbates oxidative stress in chondrocytes.

Accumulation of such cellular stresses eventually leads to pyroptosis, the inflammatory form of programmed cell death that has garnered significant attention recently for its association with various diseases [23,24,25]. This process is initiated by the recognition of DAMPs and pathogen-associated molecular patterns by pattern recognition receptors (PRRs), including nucleotide-oligomerization domains, leucine-rich repeat motifs, and pyrin domain-containing protein 3 (NLRP3). The activated PRRs induce the expression of pro-inflammatory cytokines and assemble pro-caspase-1 with apoptosis-associated speck-like protein containing a caspase recruitment domain (ASC) to form an inflammasome [26]. The inflammasome activates caspase-1 into its mature form, which in turn cleaves IL-1β, IL-18, and gasdermin D (GSDMD), converting them into their active forms [27,28]. The N-terminal fragment of GSDMD (NT-GSDMD) oligomerizes into plasma membrane pores, leading to cytolysis and release of active IL-1β and IL-18 [29]. In the cartilage under OA, excessive DAMPs are produced by synovium tissue, inducing pyroptosis of chondrocytes which causes a positive feedback loop that aggravates inflammation [30], oxidative stress, and cartilage destruction [15,31]. Recent reports have suggested that pyroptosis may serve as a potential therapeutic target for OA [32,33,34].

Flavonoids are biologically active polyphenolic compounds that are present in plants such as fruits, vegetables, and tea. It is well known that many flavonoids possess numerous health benefits, especially antioxidative and anti-inflammatory activities. Tamarixetin, also known as 4′-O-methyl quercetin, is a natural flavonoid derived from quercetin and can be isolated from certain plants, such as *Tamarix ramosissima* and *Cyperus teneriffae*. Previous studies have demonstrated its health-related effects in various models. For example, tamarixetin exhibited antioxidative activity by upregulating the Nrf2/Keap1 pathway in an in vivo model of liver disease [35]. Also, it has been reported that tamarixetin exerts anti-inflammatory effects by increasing IL-10 production and decreasing various pro-inflammatory cytokines in LPS-stimulated bone marrow dendritic cells and an in vivo mouse model [36]. However, the therapeutic potential of tamarixetin on the initiation and progression of OA has not been investigated yet. 

In this study, we aimed to evaluate the chondroprotective effects of tamarixetin in OA and elucidate the underlying molecular mechanism. Tamarixetin effectively protected chondrocytes from IL-1β-induced cellular stress by upregulating Nrf2 signaling and downregulating NF-κB signaling. Tamarixetin attenuated the OA phenotypic changes, ROS production, and pyroptosis of chondrocytes, which could result in the restoration of cartilage homeostasis. Our research proposes tamarixetin as a potential therapeutic agent against OA for the first time and simultaneously provides underlying molecular mechanisms regarding OA phenotype alleviation.

## 2. Materials and Methods

### 2.1. Cell Culture and Chondrogenic Differentiation 

Human bone-marrow-derived mesenchymal stem cells (hBMMSCs) were acquired from the American Type Culture Collection (ATCC) (Manassas, VA, USA). The cells were cultured in the stemMAC MSC expansion medium (Miltenyi Biotec, Bergisch Gladbach, Germany) supplemented with 100 units/mL penicillin and 100 μg/mL streptomycin (Gibco, Grand Island, NY, USA). At passage number 5, the cells were seeded at a density of 2 × 10^4^ cells/cm^2^ in an appropriate cell culture dish. Upon 100% confluence, chondrogenic differentiation was induced for 21 days in chondrogenic differentiation medium (CDM) containing high-glucose Dulbecco’s modified Eagle medium (DMEM) (Gibco), 100 units/mL penicillin, 100 μg/mL streptomycin, 1 mM sodium pyruvate (Welgene, Gyeongsan, Republic of Korea), 50 μg/mL ascorbic acid-2-phosphate (Sigma-Aldrich, St. Louis, MO, USA), 1% insulin–transferrin–selenium/ITS supplement (Gibco), 40 μg/mL proline (Sigma-Aldrich), 100 nM dexamethasone (Sigma-Aldrich), and 10 ng/mL rhTGF-b_3_ (R&D Systems, Minneapolis, MN, USA). CDM was replaced three times a week. The cells were cultured at 37 °C under 5% CO_2_ in a humidified atmosphere. After the chondrogenic differentiation, the cells were treated with varying amounts of tamarixetin (Sigma-Aldrich), 10 ng/mL IL-1β (SinoBiological, Beijing, China), 10 ng/mL PMA (Abcam, Cambridge, UK), and 10 μM ML385 (MedChemExpress, Princeton, NJ, USA).

### 2.2. Cell Viability Assay

Cell viability of chondrocytes was evaluated using the Quanti-Max WST-8 Cell Viability Assay Solution (Biomax, Seoul, Republic of Korea). Following the treatment of tamarixetin with or without IL-1β for 48 h, the medium was replaced with high-glucose DMEM, and the cells were incubated at 37 °C for 30 min. After incubation, the absorbance at 450 nm was measured using a Sunrise™ Absorbance microplate reader (TECAN, Männedorf, Switzerland).

### 2.3. Alcian Blue Staining

The glycosaminoglycan (GAG) content in chondrocytes was evaluated through alcian blue staining. Following the treatment with tamarixetin with or without IL-1β for 48 h, the cells were washed twice with phosphate-buffered saline (PBS; Biosolution, Seoul, Republic of Korea) and fixed by treating them with 4% formaldehyde (Sigma-Aldrich) for 15 min. The cells were then washed with PBS and stained with alcian blue staining solution (Sigma-Aldrich) for 45 min at room temperature in the dark. Next, the cells were washed twice with 3% acetic acid (Sigma-Aldrich) and imaged by microscopy (Leica, Wetzlar, Germany). For quantification of the stained GAG content, the dye was dissolved with 6% acetic acid and the absorbance at 595 nm was measured using a Sunrise™ Absorbance microplate reader.

### 2.4. Western Blot Analysis

The protein expression levels of chondrocytes were evaluated by Western blot. The cells were washed twice with PBS and lysed with RIPA buffer (Bio Solution) containing protease inhibitor (Sigma-Aldrich) and phosphatase inhibitor cocktail 2/3 (Sigma-Aldrich). The protein concentration was quantified using a Pierce™ BCA Protein Assay Kit (Thermo Fisher Scientific, Waltham, MA, USA) following the manufacturer’s instructions. Equal amounts of protein from each sample were loaded on a 10% sodium dodecyl sulfate-polyacrylamide gel for electrophoresis. The separated proteins were transferred to a polyvinylidene difluoride (PVDF) membrane (Millipore, Burlington, MA, USA). The membranes were blocked for 1 h using skim milk (BD, Franklin Lakes, NJ, USA) and incubated with primary antibodies overnight at 4 °C. After washing with 1× TBST, the membranes were incubated with secondary antibodies for 45 min at room temperature. The antibodies used in this study are listed in Table S1. The target proteins on the membrane were visualized using ECL Plus Western blotting detection reagent (Amersham Bioscience, Buckinghamshire, UK) and Chemi-Doc (Bio-Rad Laboratories, Hercules, CA, USA). The protein expression levels were quantified using Image Lab™ Software version 4.0.1 (Bio-Rad).

### 2.5. Immunofluorescence Staining

The cells were seeded on poly-L-lysine-coated coverslips. After the treatment with IL-1β and tamarixetin, the cells were washed twice with PBS and fixed by treating them with 4% formaldehyde for 15 min. The samples were then washed with PBS and permeabilized with 0.25% Triton X-100 (Sigma-Aldrich) for 10 min. To block non-specific staining, the samples were incubated with 1% bovine serum albumin (BSA) (Santa Cruz Biotechnology, Dallas, TX, USA) for 30 min. Next, the samples were treated with primary and secondary antibodies for 1 h each at room temperature. The primary antibodies used in this analysis were anti-COL2 (diluted 1:500), anti-RUNX2 (diluted 1:500), anti-Nrf2 (diluted 1:500), anti-p-NF-κB (diluted 1:200), and anti-GSDMD (diluted 1:500). The secondary antibodies used in this analysis were goat anti-rabbit IgG antibody (DyLight594) (GeneTex, Irvine, CA, USA), goat anti-rabbit Alexa 488 (Abcam). To visualize the nuclei of the cells, counterstaining with 4,6-diamidino-2-phenylindole (DAPI) (Sigma-Aldrich) was conducted. Fluorescence images were acquired with a confocal microscope (Carl Zeiss, Oberkochen, Germany) and the fluorescence intensities emitted from the markers were quantified using ImageJ version 1.53k (National Institutes of Health, Bethesda, MD, USA).

### 2.6. ROS Assay

Intracellular ROS was evaluated using fluorescent probe 2′,7′-dichlorodihydrofluorescein diacetate (DCFH-DA) (Sigma-Aldrich). The cells were seeded on poly-L-lysine-coated coverslips (22 × 22 mm). After the treatment with IL-1β and tamarixetin for 24 h, the cells were rinsed twice with PBS and stained with 20 μM of DCFH-DA in Dulbecco’s PBS (Gibco). After incubating at 37 °C for 30 min, the cells were fixed by treating them with 4% formaldehyde for 15 min. The cells were then washed with PBS and treated with 0.25% Triton X-100 for 10 min for permeabilization. The nuclei of the cells were counterstained with DAPI. Fluorescence images were acquired with a confocal microscope and the fluorescence intensity emitted by the probe was quantified using ImageJ version 1.53k.

### 2.7. Statistical Analysis

All experiments were conducted independently, and the results were expressed as mean ± standard error of the mean (SEM). The statistical significance of the data was evaluated by performing a one-way analysis of variance (ANOVA) test, followed by Bonferroni correction using GraphPad Prism 5.0 (GraphPad Software, La Jolla, CA, USA). A *p*-value of <0.05 was considered statistically significant.

## 3. Results

### 3.1. Tamarixetin Enhances Chondrogenesis in Human Chondrocytes

**Figure 1:**
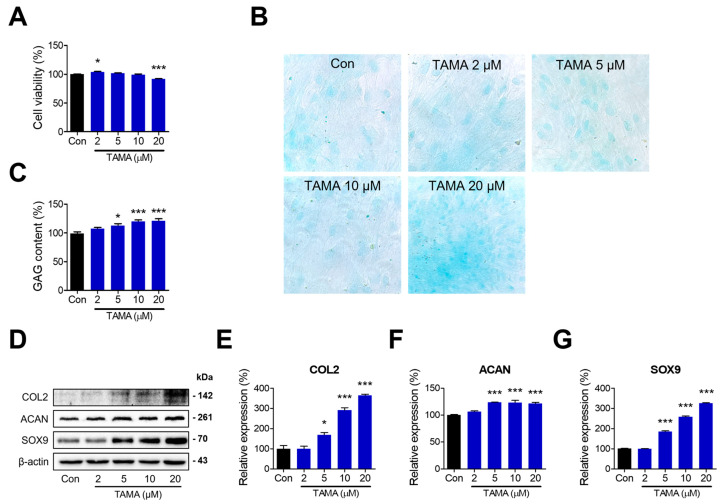
Tamarixetin treatment enhances chondrogenesis in human chondrocytes. (**A**) Relative cell viability of tamarixetin-treated chondrocytes differentiated from hBMMSC. (**B**) The GAG content was stained using Alcian Blue and observed using a light microscope (magnification: 400×). (**C**) The GAG content in chondrocytes increased when treated with tamarixetin. (**D**–**G**) Tamarixetin treatment upregulated the levels of chondrogenesis markers. The relative protein expression levels of (**E**) COL2, (**F**) ACAN, and (**G**) SOX9 were evaluated by Western blot. * *p* < 0.05 and *** *p* < 0.001, compared with the control group; TAMA: tamarixetin (2, 5, 10, and 20 µM).

To investigate the therapeutic effects of tamarixetin, chondrocytes were differentiated from hBMMSCs for 21 days in CDM. The induction of chondrogenesis was confirmed by comparing the levels of chondrogenic markers between the differentiated and undifferentiated hBMMSCs (Figure S1). The differentiated chondrocytes were treated with tamarixetin at various concentrations, and cell viability was evaluated to confirm the cytotoxicity of tamarixetin against chondrocytes. Tamarixetin did not induce observable cytotoxicity in chondrocytes, as cell viability at all treatment concentrations remained above 80% compared with the control group (Figure 1A). Next, the GAG accumulation level and protein expression levels of chondrogenic markers (COL2, ACAN, and Sry-related HMG box 9 (SOX9)) were measured following tamarixetin treatment. The GAG content was assessed via alcian blue staining, where tamarixetin significantly increased GAG content at all tested concentrations, specifically at 10 μM and 20 μM (Figure 1B,C). Furthermore, tamarixetin increased the expression of COL2, ACAN, and SOX9 above a concentration of 5 μM (Figure 1D–G). These results indicated that tamarixetin can indeed enhance the chondrogenic property of chondrocytes without exerting any observable cytotoxicity.

### 3.2. Tamarixetin Induces Chondroprotective Effects in an IL-1β-Induced OA Model

**Figure 2:**
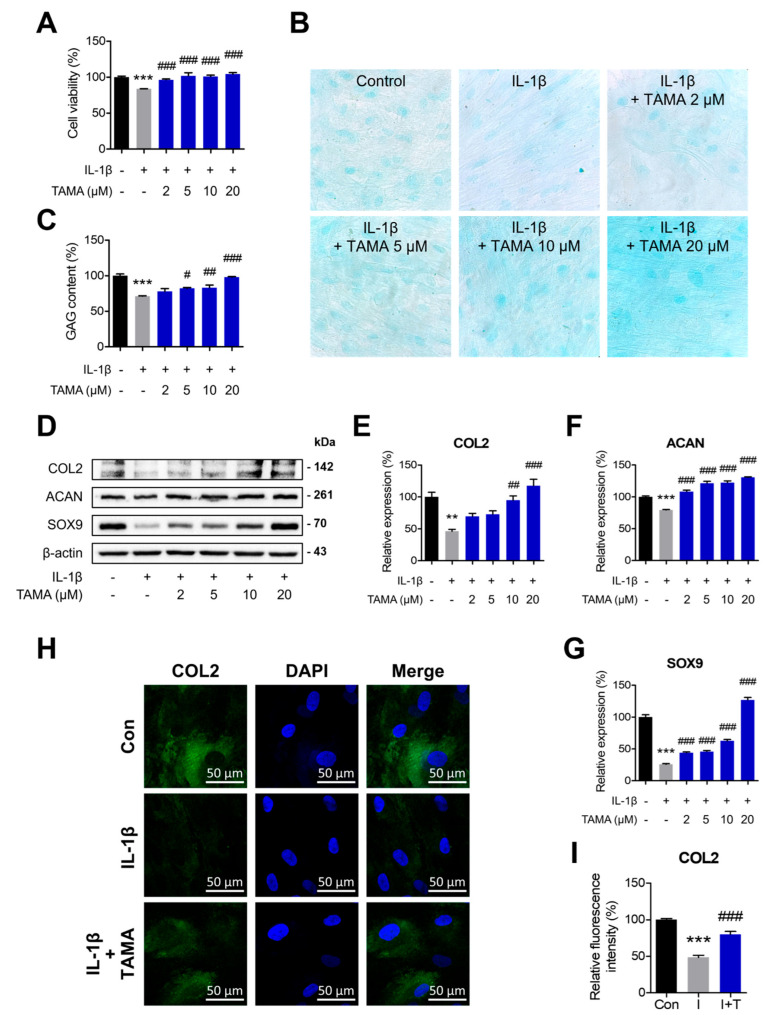
Tamarixetin exhibits chondroprotective effects in an IL-1β-induced OA model. (**A**) Relative viability of chondrocytes treated with IL-1β, with or without tamarixetin treatment for 48 h. (**B**,**C**) The GAG content normalized in IL-1β-treated chondrocytes upon tamarixetin treatment for 48 h (magnification: 400×). (**D**–**G**) The effects of tamarixetin on the expressions of chondrogenesis markers in IL-1β-treated chondrocytes. The relative protein expression levels of (**E**) COL2, (**F**) ACAN, and (**G**) SOX9 after 24 h treatment of tamarixetin and/or IL-1β were evaluated by Western blot. (**H**,**I**) Immunofluorescence analysis of COL2 expression in chondrocytes under varying treatment conditions. ** *p* < 0.01 and *** *p* < 0.001, compared with the control group; # *p* < 0.05, ## *p* < 0.01, and ### *p* < 0.001, compared with the IL-1β-treated group. IL-1β (10 ng/mL); TAMA: tamarixetin (2, 5, 10, and 20 μM); Scale bar: 50 μm.

We investigated the therapeutic effects of tamarixetin, in terms of cell viability and chondrogenic properties, in chondrocytes displaying an OA-like phenotype. The chondrocytes were treated with IL-1β to induce an OA-like phenotype. The viability of IL-1β-treated chondrocytes decreased to 83.65% when compared with the non-treated group, whereas tamarixetin treatment reversed the cytotoxicity induced by IL-1β (Figure 2A). Alcian blue staining analysis indicated that tamarixetin treatment increased the reduced GAG content in IL-1β-treated chondrocytes (Figure 2B,C). It is well known that chondrocytes in the cartilage under OA exhibit decreased expressions of chondrogenic markers. As shown in Figure 2D–G, tamarixetin treatment was observed to upregulate the protein expressions of chondrogenic markers (COL2, ACAN, and SOX9), which were reduced in the IL-1β-treated group. Furthermore, immunofluorescence analysis confirmed that tamarixetin upregulated COL2 levels in chondrocytes treated with IL-1β (Figure 2H). Collectively, it was evident that tamarixetin exerts chondroprotective effects in chondrocytes displaying an OA-like phenotype, not only increasing cell viability but also enhancing chondrogenic properties.

### 3.3. Tamarixetin Attenuates IL-1β-Induced Hypertrophic Differentiation and Dedifferentiation in Chondrocytes

**Figure 3:**
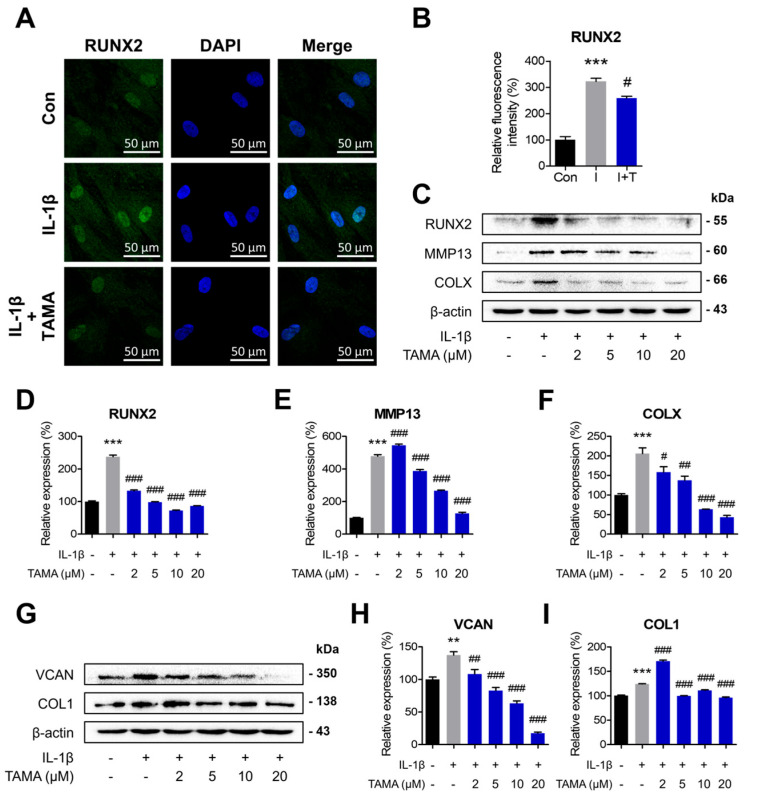
Tamarixetin attenuates IL-1β-induced hypertrophic differentiation and dedifferentiation in chondrocytes. (**A**,**B**) The relative fluorescence intensity of RUNX2 on chondrocytes was confirmed. The relative protein expression levels of (**C**–**F**) hypertrophic differentiation markers and (**G**–**I**) dedifferentiation markers after 24 h treatment of tamarixetin were evaluated by Western blot. ** *p* < 0.01 and *** *p* < 0.001, compared with the control group; # *p* < 0.05, ## *p* < 0.01, and ### *p* < 0.001, compared with the IL-1β-treated group. IL-1β (10 ng/mL); TAMA: tamarixetin (2, 5, 10, and 20 µM); Scale bar: 50 μm.

Under OA, chondrocytes lose their chondrogenic properties by undergoing hypertrophic differentiation and dedifferentiation. To determine whether tamarixetin can attenuate the IL-1β-induced hypertrophic differentiation and dedifferentiation in chondrocytes, we examined the protein expression levels of specific markers through Western blot. Treatment of IL-1β upregulated both the expressions of hypertrophic markers (runt-related transcription factor 2 (RUNX2), MMP13, and COLX) and dedifferentiation markers (versican (VCAN) and COL1). The increased levels of hypertrophic markers in the IL-1β-treated group were significantly downregulated by tamarixetin treatment, except for the expression of MMP13 at 2 μM tamarixetin (Figure 3C–E). Immunofluorescence staining was conducted to further analyze the protein expression level and translocation of RUNX2, a major transcriptional factor of hypertrophic differentiation (Figure 3A). Tamarixetin reduced RUNX2 expression and nuclear translocation compared with the IL-1β-treated group. In the case of dedifferentiation, tamarixetin reduced the protein expression of VCAN in a dose-dependent manner (Figure 3F,G). The protein expression of COL1 was also observed to be reduced following tamarixetin treatment, except at 2 μM (Figure 3F,H). Taken together, tamarixetin was observed to reduce the IL-1β-induced OA phenotypic changes, including hypertrophic differentiation and dedifferentiation. As the results demonstrated that 20 μM tamarixetin effectively attenuates IL-1β-induced dysfunction in chondrocytes, subsequent experiments were conducted at this concentration.

### 3.4. Tamarixetin Attenuates IL-1β-Induced ROS by Activating the Nrf2/HO-1 Signaling Pathway in Chondrocytes

**Figure 4:**
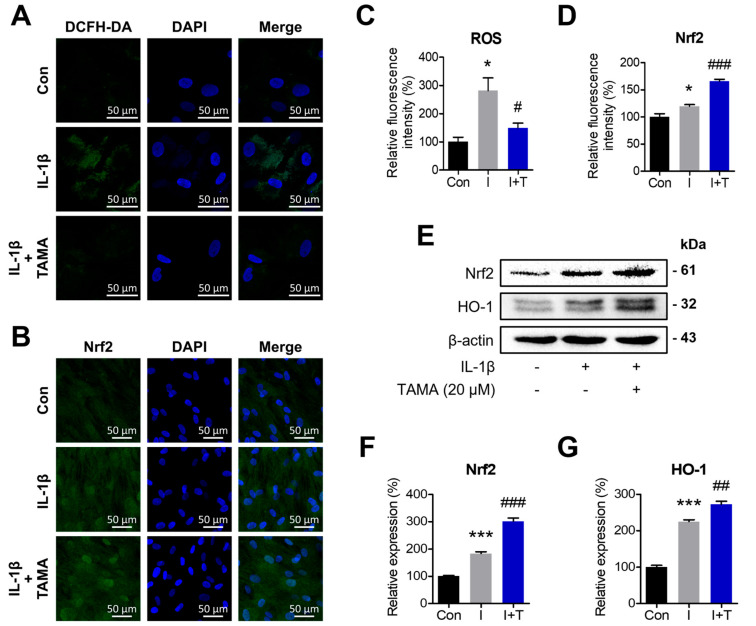
Tamarixetin attenuates IL-1β-induced ROS production by activating the Nrf2/HO-1 signaling pathway in chondrocytes. The cells were treated with tamarixetin for 2 h for the experiments. The relative levels of (**A**,**C**) ROS production and (**B**,**D**) Nrf2 expression in chondrocytes as evaluated by DCFH-DA and immunofluorescence staining. (**E**–**G**) The relative protein expression levels of Nrf2 and HO-1 were evaluated by Western blot. * *p* < 0.05 and *** *p* < 0.001 compared with the control group; # *p* < 0.05, ## *p* < 0.01, and ### *p* < 0.001 compared with the IL-1β-treated group. Con: Control; I: IL-1β (10 ng/mL); T: tamarixetin (20 µM); Scale bar: 50 μm.

Oxidative stress is known to be directly associated with dysfunction in chondrocytes. The highly expressed inflammatory mediators, such as IL-1β, mediate ROS production and contribute to OA progression. To elucidate the antioxidative effects of tamarixetin in IL-1β-treated chondrocytes, we evaluated the ROS production level and expressions of Nrf2 and heme oxygenase 1 (HO-1), the proteins that regulate antioxidative processes. DCFH-DA staining showed that tamarixetin treatment can significantly reduce the ROS production in IL-1β-treated chondrocytes (Figure 4A). The protein expressions of Nrf2 and HO-1 were increased upon IL-1β treatment, where tamarixetin further elevated these expressions (Figure 4B,D–G). These results suggested that tamarixetin attenuates the IL-1β-induced oxidative stress in chondrocytes by activating the Nrf2/HO-1 signaling pathway.

### 3.5. Tamarixetin Inhibits the NF-κB and MAPK Signaling Pathways in an IL-1β-Induced OA Model

**Figure 5:**
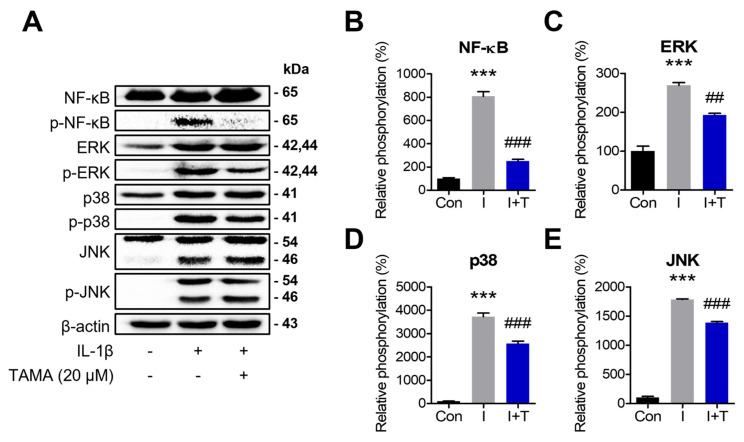
Tamarixetin inhibits the NF-κB and MAPK signaling pathways in an OA model. (**A**–**E**) The inhibitory effects of tamarixetin on the expression and phosphorylation of NF-κB, ERK, JNK, and p38 were evaluated by Western blot. *** *p* < 0.001, compared with the control group; ## *p* < 0.01 and ### *p* < 0.001 compared with the IL-1β-treated group. Con: Control; I: IL-1β (10 ng/mL); T: tamarixetin (20 µM).

IL-1β rapidly activates the MAPK and NF-κB signaling pathways and contributes to inflammation and ROS production. We investigated whether tamarixetin could inhibit the protein expressions and phosphorylation of NF-κB and MAPKs (including extracellular signal-regulated kinase (ERK), c-Jun N-terminal kinase (JNK), and p38) by Western blot. IL-1β treatment was observed to activate NF-κB and MAPK in chondrocytes, with NF-κB phosphorylation reaching its peak level at 10 min post-treatment and at 20 min post-treatment for ERK and JNK (Figure S2). In the case of p38, it was observed to be highly phosphorylated at 10, 20, and 30 min post-treatment. Therefore, we treated with IL-1β with or without tamarixetin for 10 min to evaluate the activity of NF-κB and for 20 min to assess MAPK activity. IL-1β significantly induced the phosphorylation of NF-κB and MAPK, which was attenuated by tamarixetin treatment (Figure 5). These results indicated that tamarixetin inhibit the activation of NF-κB and MAPK signaling pathways induced by IL-1β in chondrocytes.

### 3.6. Tamarixetin Protects Chondrocytes from IL-1β-Induced Pyroptosis

**Figure 6:**
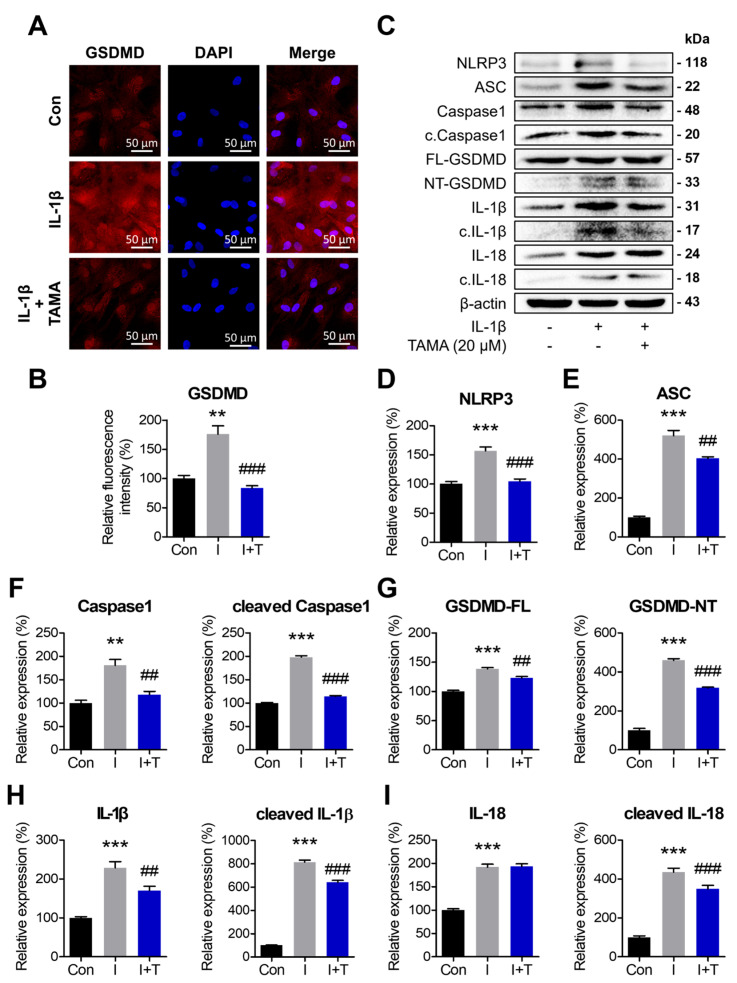
Tamarixetin protects chondrocytes from IL-1β-induced pyroptosis. The cells were treated with tamarixetin for 48 h for all experiments. (**A**,**B**) The relative levels of GSDMD in chondrocytes as analyzed by immunofluorescence staining. (**C**–**I**) The relative protein expression levels of pyroptosis markers were evaluated by Western blot. ** *p* < 0.01 and *** *p* < 0.001, compared with the control group; ## *p* < 0.01 and ### *p* < 0.001 compared with the IL-1β-treated group. Con: Control; I: IL-1β (10 ng/mL); TAMA and T: tamarixetin (20 µM); Scale bar: 50 μm.

Pyroptosis contributes to the exacerbation of inflammation and OA severity. Inhibiting pyroptosis-associated components has gained attention for its potential to attenuate OA. Therefore, we evaluated the levels of pyroptotic proteins in chondrocytes displaying an OA phenotype with or without tamarixetin treatment. IL-1β treatment was observed to upregulate the inflammasome-related proteins (NLRP3 and ASC), maturation of caspase-1, and cleavage of GSDMD, IL-1β, and IL-18. Notably, tamarixetin treatment mitigated these effects (Figure 6C–H). The expression of GSDMD, which contributes to the pyroptotic pore formation in the plasma membrane, was visualized through immunofluorescence staining (Figure 6A). These results implied that tamarixetin disrupts the vicious cycle of escalating OA by inhibiting pyroptosis in OA chondrocytes.

### 3.7. Recovery of Nrf-2 Activity and Inhibition of NF-κB Activity by Tamarixetin Treatment Suppress Pyroptosis in an IL-1β-Induced OA Model

**Figure 7:**
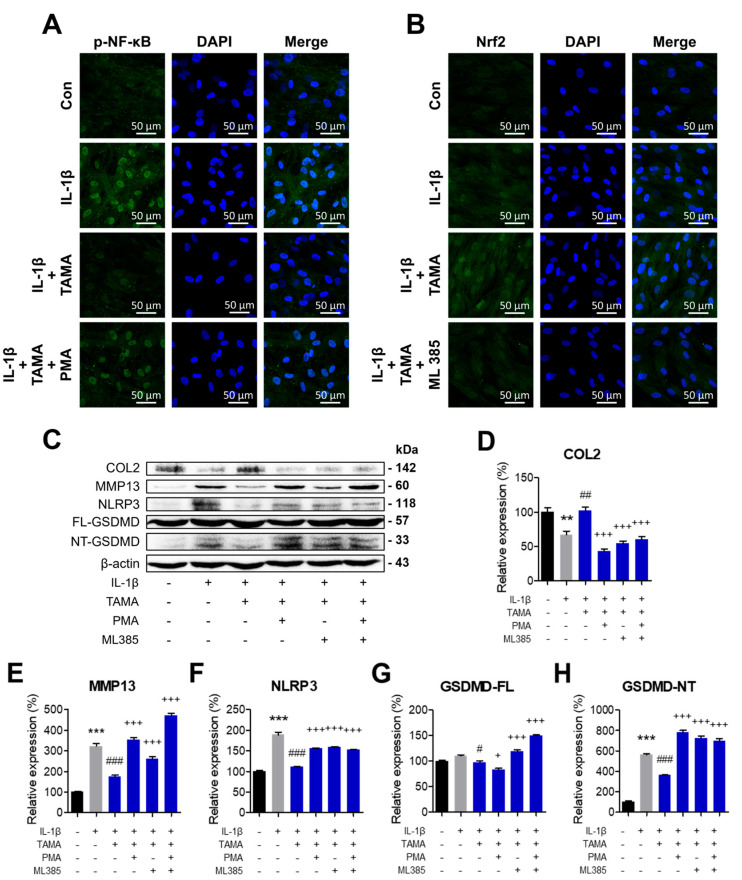
Tamarixetin treatment suppressed pyroptosis and OA progression in an IL-1β-induced OA model by restoring Nrf-2 activity and inhibiting NF-κB activity. (**A**,**B**) The relative levels of NF-κB and Nrf2 in chondrocytes under different treatment conditions, as analyzed by immunofluorescence staining. (**C**–**H**) The relative protein expression levels of COL2, MMP13, and pyroptosis markers were evaluated by Western blot. ** *p* < 0.01 and *** *p* < 0.001, compared with the control group; # *p* < 0.05, ## *p* < 0.01, and ### *p* < 0.001 compared with the IL-1β-treated group; + *p* < 0.05 and +++ *p* < 0.001 compared with the IL-1β/tamarixetin co-treated group. Con: Control; I: IL-1β (10 ng/mL); TAMA: tamarixetin (20 µM).

NF-κB is a transcription factor that upregulates the expression of NLRP3 and induces pyroptosis. Additionally, oxidative stress is widely acknowledged as a major inducer of pyroptosis. Thus, inhibiting NF-κB and promoting antioxidative pathways are potentially promising therapeutic approaches to inhibit pyroptosis and attenuate OA pathology. As we demonstrated that tamarixetin treatment effectively enhances the Nrf2/HO-1 signaling pathway and reduces the activation of NF-κB and pyroptosis in chondrocytes, we further investigated the mechanism by which tamarixetin inhibits pyroptosis. In chondrocytes co-treated with IL-1β and tamarixetin, NF-κB activation and Nrf2 suppression were observed, as evaluated using phorbol 12-myristate 13-acetate (PMA) and MHY385, respectively. Immunofluorescence analysis confirmed that PMA successfully upregulated the protein expression and translocation of NF-κB (Figure 7A), while MHY385 downregulated those of Nrf2 (Figure 7B). The OA-like phenotype alleviated by tamarixetin was observed to be re-exacerbated by PMA and/or MHY385 treatment, as demonstrated by the reduced level of anabolism, along with re-stimulated pyroptosis and catabolism (Figure 7C–H). Collectively, it was concluded that tamarixetin can attenuate OA progression in IL-1β-treated chondrocytes by both inhibiting NF-κB activation and upregulating Nrf2 signaling.

## 4. Discussion

OA is the most prevalent form of arthritis, affecting about 7% of the world’s population [2]. However, due to the lack of available treatment options, numerous OA patients suffer from mobility impairment and severe pain. As such, the FDA has classified OA as a serious disease that requires accelerated treatment development [37]. The main characteristics of OA include cartilage destruction, calcification, and synovial inflammation; however, the detailed molecular mechanisms underlying its pathology are not yet fully elucidated. This study focused on investigating the protective effects of tamarixetin in chondrocytes showing an OA-like phenotype and elucidating its mechanism.

Flavonoids are naturally occurring compounds found in plants, and they are widely known for their beneficial health properties [38]. Numerous studies have demonstrated the therapeutic effects of flavonoids, including antioxidative, anti-inflammatory, and anti-apoptotic properties, which are closely related to OA pathology [39]. Among these compounds, quercetin and its derivates have shown potential in preventing and treating various chronic inflammatory diseases [40]. Tamarixetin is a derivative of quercetin with methylation at the 4′ carbon position. It is known to exert significantly elevated anti-inflammatory effects compared with other quercetin derivatives [41]. Additionally, it has been reported that tamarixetin regulates the NF-κB [42] and JNK [36,43] signaling pathways, which play crucial roles in OA pathology by binding to the ATP-binding sites of IκB kinase 2 and JNK1. Therefore, for the first time, we explored the therapeutic potential of tamarixetin in chondrocytes displaying an OA phenotype in this study.

BMMSCs are progenitor cells capable of differentiating into chondrocytes in human articular cartilage. Once cartilage is damaged due to injury or aging, it cannot repair itself due to its limited regenerative capacity, which may lead to OA development [44,45]. For the recovery of cartilage tissue, the recruitment of progenitor cells and the chondrogenesis of local or migrated BMMSCs are necessary. During the chondrogenesis in hBMMSCs, the SOX9 transcriptional factor is expressed, which induces the transcriptional activation of the chondrogenic pathway, including the expressions of cartilage matrix proteins (COL2 and ACAN). As we used hBMMSCs as chondroprogenitor cells in this study, we first confirmed whether hBMMSCs can be fully differentiated into chondrocytes after 21 days of culture in CDM. The cluster of differentiation 14 (CD14), a surface marker of chondrocytes, was found to be highly expressed after this chondrogenic induction (Figure S1) [46,47]. Also, the protein expression levels of COL2, ACAN, and SOX9 were upregulated with CDM treatment, confirming the successful differentiation of hBMMSC into chondrocytes.

To explore the chondroprotective effects of tamarixetin against OA pathology, we next evaluated the enhancement and restoration of chondrogenesis following tamarixetin treatment in both healthy and IL-1β-treated chondrocytes. At various concentrations of tamarixetin, both GAG content and chondrogenic marker expressions increased in both IL-1β-treated and untreated chondrocytes (Figure 1 and Figure 2). GAGs, also known as mucopolysaccharides, are major components of the cartilage that facilitate smooth joint movement and pressure absorption. A reduction in GAG content indicates chondrocyte dysfunctions and is utilized as a marker of OA [48]. Our results showed that tamarixetin treatment can increase the GAG content in chondrocytes in a dose-dependent manner, achieving 120.861% of the basal level (control) when treated at 20 μM. Following IL-1β treatment, the GAG content decreased to 71.103% of that of the control group. Notably, 20 μM tamarixetin could restore the content to 97.929% (Figure 2B,C). These results suggested that tamarixetin can protect chondrocytes from structural deterioration and altered homeostasis induced by IL-1β.

The inflammatory responses and associated biological processes play pivotal roles in the progression of OA [49]. Inflammatory mediators are primarily produced by synovitis, inflammation in the synovial membrane, which activates chondrocytes through the synovial fluid [50,51]. In the activated chondrocytes, the balance between anabolism and catabolism is disrupted, which is critical for maintaining cartilage homeostasis. In healthy cartilage, chondrocytes maintain the elasticity of the cartilage microenvironment by expressing cartilage-specific ECM, such as COL2 and ACAN. However, in the cartilage under OA, chondrocytes undergoing hypertrophic differentiation or dedifferentiation alter the existing cartilage ECM by expressing proteolytic enzymes. OA chondrocytes also drastically modify the cartilage matrix, making it more fibrotic and stiffer through the secretion of COLX, COL1, and VCAN. Thus, preventing such phenotypical changes in chondrocytes is crucial for treating OA pathology. In this study, tamarixetin treatment was shown to downregulate the expressions of both hypertrophic and dedifferentiation markers that were elevated by IL-1β treatment (Figure 3). In the case of RUNX2, a transcriptional factor that positively regulates the hypertrophic genes, tamarixetin treatment decreased its protein expression and translocation to the cell nucleus. This suggests that the compound has ameliorative effects against IL-1β-induced phenotypic changes at the transcriptional level [52].

It is widely recognized that, along with inflammation, oxidative stress is closely related to chondrocyte dysfunctions [22]. In healthy chondrocytes, ROS are used for cellular functions such as signaling and maintenance of homeostasis, while remaining at non-toxic levels. In contrast, OA pathology produces excessive and toxic levels of ROS, in which the resulting imbalance between ROS and antioxidants accelerates inflammation [53]. Under such circumstances, the cellular defense mechanism against oxidative stress activates Nrf2 to maintain redox homeostasis and removes the ROS molecules from the affected cells. We observed that tamarixetin treatment can effectively reduce the level of ROS production by upregulating the Nrf2/HO-1 antioxidative signaling pathway (Figure 4). Furthermore, our results indicated that the activation of the Nrf2 pathway upon IL-1β stimulation is further enhanced by tamarixetin treatment, thereby boosting the cellular antioxidative system.

When IL-1β binds to IL-1R, the myeloid differentiation primary response 88-dependent pathway can activate both the MAPK and NF-κB signaling pathways, which can exacerbate inflammation and dysfunction of chondrocytes [54]. Since these pathways can be activated concurrently, we evaluated the time-dependent phosphorylation of MAPK and NF-κB upon IL-1β treatment. The results showed that the phosphorylation of MAPK and NF-κB reached their peaks at 20 min and 10 min post-treatment, respectively; these timepoints were accordingly used for tamarixetin treatment timepoints (Figure S2 and Figure 5). Tamarixetin treatment was observed to significantly suppress the activation of MAPK and NF-κB, contributing to the reduction of inflammatory responses. 

NF-κB and Nrf2 are major transcription factors that regulate inflammatory and antioxidative pathways, respectively. These pathways are closely related, and their balance is crucial for maintaining homeostasis in response to cellular stresses in chondrocytes. Under OA, increased oxidative stress and pro-inflammatory signals disrupt this balance, ultimately leading to cell death processes, such as apoptosis and pyroptosis [22]. Aggravated oxidative stress and increased DAMPs in the cartilage under OA activate the NF-κB pathway, triggering the assembly of NLRP3 inflammasome and pyroptotic cell death in chondrocytes. Therefore, we explored whether tamarixetin can attenuate the NLRP3 inflammasome-dependent pyroptosis induced by IL-1β (Figure 6). We observed that tamarixetin can indeed inhibit pyroptosis via both NF-κB inhibition and Nrf2 activation. Here, the presence of NF-κB activator and/or Nrf2 inhibitor prevented the restoration of chondrogenic properties and inhibition of IL-1β-induced pyroptosis by tamarixetin treatment (Figure 7). Similar to our results, it was reported that several phytochemicals showed chondroprotective effects through NF-κB inhibition or Nrf2 activation. For example, morroniside [32] and loganin [55] attenuate pyroptosis and OA progression by inhibiting NF-κB signaling. Wogonin, a plant-derived small molecule, reduced oxidative stress, inflammation, and matrix degradation in OA chondrocytes by activating Nrf2 signaling pathways [56]. In the case of quercetin, a chemical precursor of tamarixetin, it exhibited anti-arthritic effects in several in vitro and in vivo experiments, including inhibiting NF-κB pathways [57] and pro-inflammatory cytokine-induced NLRP3 activation [58]. Parallel with the previous results, the current study demonstrates the molecular mechanism of tamarixetin in attenuating cellular stress in chondrocytes, highlighting the therapeutic potential of flavonoids in treating OA phenotype.

Taken together, this study revealed tamarixetin’s efficacy in attenuating the IL-1β-induced OA phenotype, oxidative stress, and pyroptosis in chondrocytes. We confirmed that these protective effects are mediated by the inhibition of the NF-κB pathway and activation of the Nrf2 pathway. Since our research investigated the effects of tamarixetin on chondrocytes differentiated from hBMMSCs, further investigations using primary cells or in vivo models may be crucial to fully understand the complex nature of OA in a physiological context. Additionally, exploring tamarixetin’s effects on pain modulation and metabolic changes within the OA microenvironment could broaden the scope of phytochemicals in treating OA. In conclusion, these findings not only propose tamarixetin as a promising therapeutic agent for alleviating OA phenotype but also provide insights into associated molecular mechanisms of the flavonoid.

## 5. Conclusions

The study aimed to explore the potential of tamarixetin as a therapeutic agent against OA phenotype by demonstrating its capacity to protect chondrocytes from IL-1β-induced cellular stress. Tamarixetin exhibited a chondroprotective effect by restoring chondrogenic differentiation while enhancing the Nrf2 antioxidative pathway. Also, it attenuated inflammation and resulting pyroptotic cell death by suppressing MAPK/NF-κB signaling pathways. These findings suggest tamarixetin as a promising candidate for future OA therapies and emphasize the broader therapeutic application of phytochemicals in managing degenerative joint diseases including OA.

## Data Availability

The original contributions presented in the study are included in the article/Supplementary Materials, further inquiries can be directed to the corresponding author (Jung-Suk Sung).

## Supplementary Materials

The following supporting information can be downloaded at: https://www.mdpi.com/article/10.3390/antiox13101166/s1, Figure S1: Construction of an in vitro monolayer chondrogenesis model using hBMMSC; Figure S2: Time course changes in NF-κB/MAPK signaling; Table S1: List of antibodies used in the study.
