# Supplementary material for: Tamarixetin Protects Chondrocytes against IL-1β-Induced Osteoarthritis Phenotype by Inhibiting NF-κB and Activating Nrf2 Signaling
Source: Antioxidants (Basel). 2024 Sep 25;13(10):1166. doi: 10.3390/antiox13101166 (PMC11505541; doi:10.3390/antiox13101166)
Supplement: Supplementary file 1 [file antioxidants-13-01166-s001.zip › antioxidants-3198662-supplementary.pdf]

Supplementary Materials

Tamarixetin protects chondrocytes against osteoarthritis by inhibiting NF-κB and activating Nrf2 signaling

Table S1. List of antibodies used in the study.

Figure S1. Construction of an in vitro monolayer chondrogenesis model using hBMMSCs.

Figure S2. Time course changes in NF-κB/MAPK signaling.

Table S1. List of antibodies used in the study.

Target	Catalogue number	Source	Dilution
CD14	sc-58951	Santacruz	Western blot - 1:500
COL2	ab34712	Abcam	Western blot - 1:500<br>ICC - 1:500
ACAN	GTX03179	Genetex	Western blot - 1:500
SOX9	82630S	Cell Signaling Technology	Western blot - 1:500
RUNX2	sc-10758	Santacruz	Western blot - 1:500<br>ICC - 1:500
MMP13	sc-515284	Santacruz	Western blot - 1:500
COLX	ab182563	Abcam	Western blot - 1:500
COL1	Ab34710	Abcam	Western blot - 1:500
VCAN	MA5-27638	Invitrogen	Western blot - 1:500
Nrf2	A11159	Abclonal	Western blot - 1:500<br>ICC - 1:500
HO-1	sc-136960	Santacruz	Western blot - 1:500
NF-κB	ab16502	Abcam	Western blot - 1:500
ERK 1/2	9107S	Cell Signaling Technology	Western blot - 1:500
p38	9212S	Cell Signaling Technology	Western blot - 1:500
JNK	9252s	Cell Signaling Technology	Western blot - 1:500
p-NF-κB	sc-136548	Santacruz	Western blot - 1:500<br>ICC - 1:200

p-ERK	sc-7383	Santacruz	Western blot - 1:500
p-p38	4511S	Cell Signaling Technology	Western blot - 1:500
p-JNK	9255S	Cell Signaling Technology	Western blot - 1:500
NLRP3	A5652	Abclonal	Western blot - 1:500
ASC	13833S	Cell Signaling Technology	Western blot - 1:500
Caspase1	PA5-29324	Invitrogen	Western blot - 1:500
Cleaved caspase1	4199S	Cell Signaling Technology	Western blot - 1:500
GSDMD	20770-1-AP	Proteintech	Western blot - 1:500<br>ICC - 1:500
IL-1β	P420B	Invitrogen	Western blot - 1:500
IL-18	PA5-110679	Invitrogen	Western blot - 1:500
β-actin	sc-47778	Santacruz	Western blot - 1:500
Anti-rabbit IgG HRP-linked antibody	7074S	Cell Signaling Technology	Western blot - 1:1500
m-IgGκ BP-HRP	sc-516102	Santacruz	Western blot - 1:1500
Goat anti-rabbit IgG antibody (DyLight594)	GTX213110	Genetex	ICC - 1:1000
Goat anti-rabbit Alexa 488	ab181448	Abcam	ICC - 1:1000
Anti-Mouse IgG FAB2 Alexa 488 Fluor	4408S	Cell Signaling Technology	ICC - 1:1000

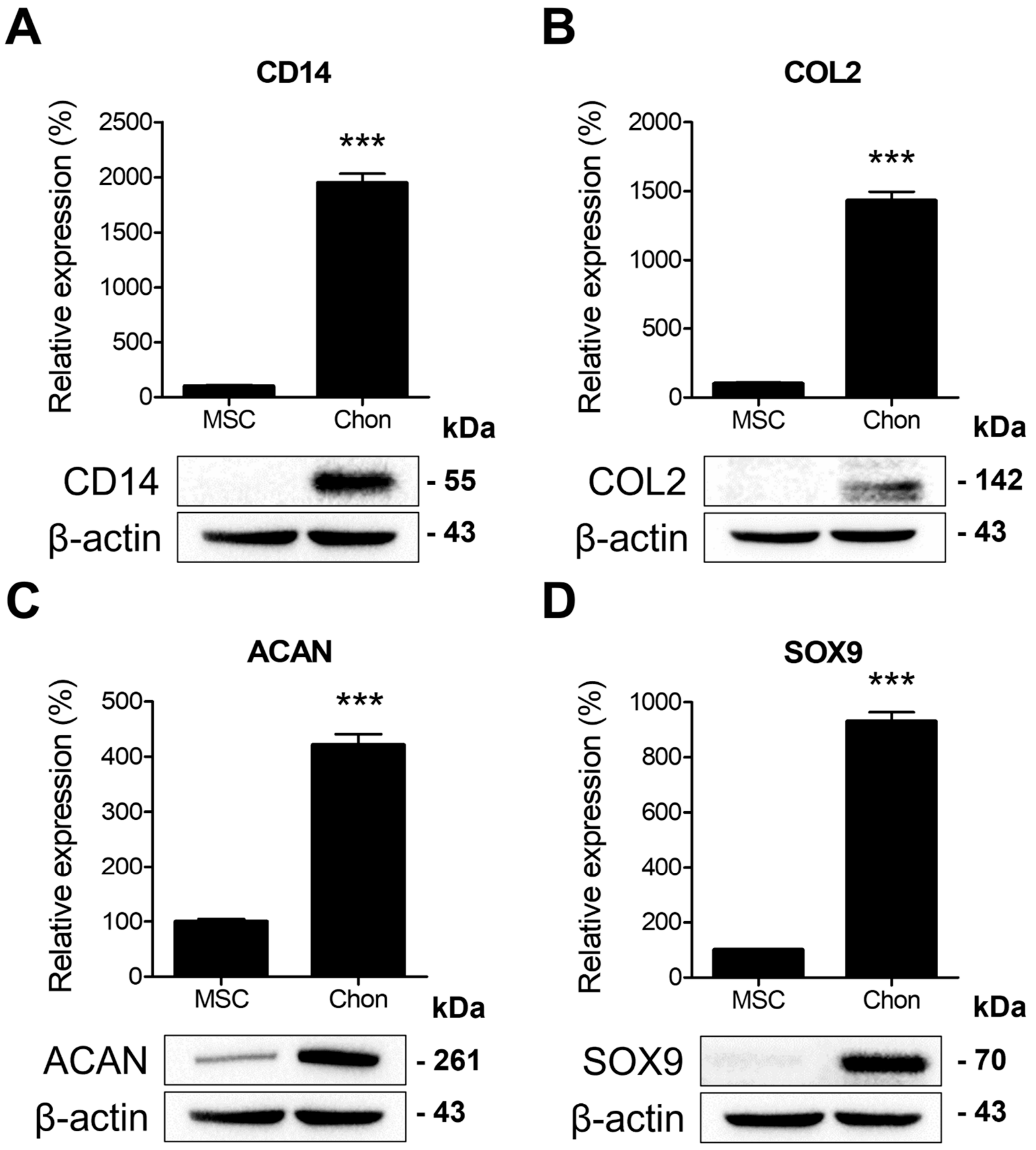

Figure S1. Construction of an in vitro monolayer chondrogenesis model using hBMMSCs. hBMMSCs were differentiated from hBMMSCs for 21days in CDM. Relative protein expression levels of (**A**) CD1 4, (**B**) COL2, (**C**) ACAN, and (**D**) SOX9 were evaluated through western blot analysis. *** $p < 0.001$, co mpared with the hBMMSCs group; MSC: hBMMSCs; Chon: chondrocytes.

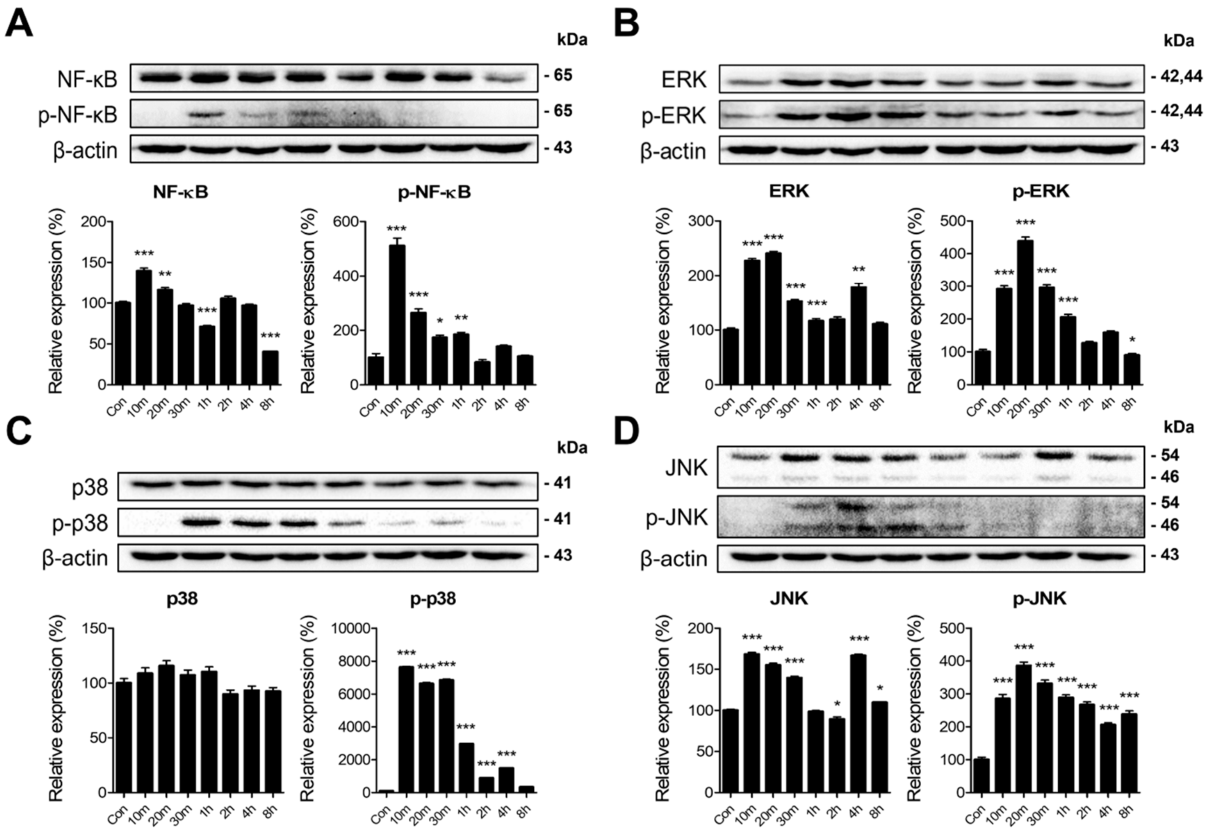

Figure S2. Time course changes in NF-κB/MAPK signaling. Chondrocytes were treated with IL-1β for 10 min, 20 min, 30 min, 1, 2, 4, and 8 h for the experiment. Relative protein expression levels of (**A**) NF-κB, (**B**) ERK, (**C**) p38, and (**D**) JNK were evaluated through western blot analysis. . * $p < 0.05$, ** $p < 0.01$, and *** $p < 0.001$, compared with the control group. Con: Control; IL-1β: 10 ng/mL.
